# 574. Antibodies Targeting *Candida albicans* Als3 and Hyr1 Antigens Protect Neonatal Mice from Candidiasis

**DOI:** 10.1093/ofid/ofac492.627

**Published:** 2022-12-15

**Authors:** Shakti Singh, Sunna Nabeela, Ashley M Barbarino, Ashraf S Ibrahim, Priya Uppuliri

**Affiliations:** The Lundquist Institute at Harbor-UCLA Medical Center, Torrance, California; The Lundquist Institute at Harbor-UCLA Medical Center, Torrance, California; The Lundquist Institute at Harbor-UCLA Medical Center, Torrance, California; The Lundquist Institute at Harbor-UCLA Medical Center, Torrance, California; The Lundquist Institute at Harbor-UCLA Medical Center, Torrance, California

## Abstract

**Background:**

Pre-term infants in neonatal intensive care units are vulnerable to fungal sepsis. In this patient population, *C. albicans* remains the predominant fungal pathogen causing high morbidity and mortality, despite antifungal therapy. Thus, new therapeutic strategies against neonatal candidiasis are needed. We have reported that vaccination with *C. albicans* cell wall N-termini recombinant proteins Als3 (rAls3p-N) and Hyr1 (rHyr1p-N) protected adult mice from disseminated candidiasis. Further, NDV-3A (rAls3p-N+alum) protected women from recurrent vulvovaginal candidiasis, which was correlated with anti-Als3p IgG2 isotype titer. Here, we evaluated the efficacy of Als3p and Hyr1p based vaccine in a neonatal candidiasis mouse model.

**Methods:**

Inbred BALB/c mice strain was used for both active and passive vaccination studies. Female 4-6 weeks old mice were vaccinated with rAls3p-N or rHyr1p-N antigens mixed with complete Freund’s adjuvant (CFA, priming)/incomplete Freund’s adjuvant (IFA, boosting). Mice were mated after the boost and pups (3 days old) born to vaccinated mice were infected intraperitoneally (IP) with *C. albicans*. For passive vaccination, 3 days old naive pups were IP infected with *C. albicans* and treated with serum obtained from vaccinated adult mice. Fungal burdens were determined in the kidneys of infected neonate mice at 3 days post-infection. Antibody titers were determined by ELISA.

**Results:**

CFA/IFA formulated Als3 and Hyr1 vaccines induced a robust antibody response with a ten-fold higher titer of IgG2, than attained by either antigen formulated with alum. Transplacental transfer of these antibodies significantly reduced the fungal burden in the kidneys of mice pups, and adoptive transfer of vaccinated mothers’ sera into pups displayed similar levels of protection. Neutrophils were found important for this efficacy. Anti-Hyr1 antisera potentiated the activity of fluconazole in protecting from *C. albicans* infection.

**Conclusion:**

Our current studies are the first to emphasize the importance of anti-Als3 and anti-Hyr1 antibodies in preventing neonatal candidiasis. Considering that Candida infections in low birth weight infants is a lethal infection, active and passive vaccination strategies using these antigens could have profound clinical significance.

**Disclosures:**

**All Authors**: No reported disclosures.

